# Audiovestibular Disorders after COVID-19 Vaccine: Is There an Association?

**DOI:** 10.3390/audiolres12030024

**Published:** 2022-04-21

**Authors:** Davide Pisani, Federico Maria Gioacchini, Pasquale Viola, Alfonso Scarpa, Alessia Astorina, Massimo Re, Gianmarco Marcianò, Francesco Manti, Roberta Anzivino, Giuseppe Chiarella

**Affiliations:** 1 Unit of Audiology, Regional Centre of Cochlear Implants and ENT Diseases, Department of Experimental and Clinical Medicine, Magna Graecia University, 88100 Catanzaro, Italy; davidepisani@gmail.com (D.P.); alessiaastorina7@gmail.com (A.A.); chiarella@unicz.it (G.C.); 2 Ear, Nose, and Throat Unit, Department of Clinical and Molecular Sciences, Polytechnic University of Marche, Via Conca 71, 60020 Ancona, Italy; giox83@hotmail.com (F.M.G.); remassimo@hotmail.com (M.R.); 3 Department of Medicine and Surgery, University of Salerno, 84084 Fisciano, Italy; alfonsoscarpa@yahoo.it; 4 Department of Health Science, University Magna Graecia, 88100 Catanzaro, Italy; gianmarco.marciano@libero.it; 5 U.O.C. Radiodiagnostics, Magna Graecia University, 88100 Catanzaro, Italy; mantifra@unicz.it; 6 Otolaryngology Unit, Di Venere Hospital, 70131 Bari, Italy; roberta.anzivino@gmail.com

**Keywords:** COVID-19, vaccine, adverse event, SSNHL, tinnitus, vertigo, dizziness, AEFI

## Abstract

The SARS-CoV-2 vaccination campaign is probably one of the most historic public hygiene measures in modern medicine. The drama of the pandemic has forced the scientific community to accelerate the development and commercialization of vaccines, thereby enhancing the phases of active surveillance. Among the adverse events following immunization (AEFI) reported, those of an audiovestibular interest, such as sudden sensorineural hearing loss (SSNHL), tinnitus, dizziness, and vertigo, constitute a very small percentage. There are many plausible etiological hypotheses, and scientific research needs to pay more attention to the correct collection of data, which up until now have often been inadequate and fragmented, on which to base future studies. SSNHL, new onset tinnitus, vertigo, and dizziness require a prompt evaluation, while the proposed treatment is the same as it is for events unrelated to vaccination. These are uncommon adverse events, and the risk rates for these diseases have not increased in conjunction with the COVID-19 vaccinations, therefore there is no justification of any hesitation towards the vaccination campaign.

## 1. Introduction

The COVID-19 pandemic, caused by severe acute respiratory syndrome coronavirus 2 (SARS-CoV-2), was a massive global health concern which had a devastating impact on public health, economy, and social life worldwide. The COVID-19 phenotype is highly heterogeneous: it ranges from asymptomatic to severe and/or with fatal manifestations. The effectiveness of the immune system is the key to overcoming the disease. Unfortunately, hyperactivated immune responses, which can lead to respiratory insufficiency and other complications such as thrombotic or thromboembolic events, are not uncommon, given the possibility of SARS-CoV-2 to activate both the innate and acquired immune response [1].

The first months of the pandemic forced drastic containment measures, such as lockdowns due to the limited effectiveness of the proposed therapies [2,3]. Consequently, the entire scientific community focused on the development of vaccines capable of preventing severe forms of the disease.

Since December 2020, less than a year from the first reported case of COVID-19, several vaccines against SARS-CoV-2 have been authorized for emergency use [4].

In total, four types of COVID-19 vaccines were released: whole virus (live attenuated, inactivated), nucleic acid (mRNA, DNA), viral vector (non-replicating, replicating), and protein-based (subunit, virus-like particle) vaccines [5].

Whole virus vaccines are based on a weakened or inactivated phenotype of SARS-CoV-2 to trigger immunity; the nucleic acid vaccines introduce mRNA or DNA into the cells in order to induce production of antibodies against the SARS-CoV-2 spike protein; viral vector vaccines use a chemically weakened virus (e.g. adenovirus) to insert the code for SARS-CoV-2 antigens into the cells; and protein subunit vaccines are based on the spike protein or its antigenic fragments [6].

Vaccines undergo a long process of validation to ensure their efficacy and safety before being administered to a large-scale population. This process requires randomized, controlled, double-blind clinical trials, as well as a precise comparison of incidence of adverse events between the study-group and the placebo-group, all of which takes a long time, in order to demonstrate the effectiveness and safety [7].

The worsening of global development during the COVID-19 pandemic placed pressure on shortening this process to introduce possible life-saving vaccines; starting from December 2020, the first emergency approval was granted in the United Kingdom and the United States, shortly followed by Europe [8,9,10].

In this scenario, widespread use of the adverse event monitoring network is mandatory.

These systems allow healthcare professionals and citizens to report any adverse reactions on a database that is made available, guaranteeing continuous monitoring and study opportunities for the scientific community.

To encourage and speed up herd immunization, many nations have introduced mandatory vaccination, as well as highly restrictive measures for the unvaccinated [11].

All of the above gave rise to an anti-vaccination feeling that has amplified the debate on the adverse events of vaccination [12].

Vaccines are closely monitored to collect data on any adverse effects. Europe monitors AEFI through the European Medicine Agency (EMA) [13] and collects data in a database called EudraVigilance [14], while the Food and Drug Administration (FDA) in the USA, monitors COVID-19 vaccines in collaboration with the Centers for Disease Control and Prevention (CDC) and other healthcare data systems [15].

The data from vaccine monitoring systems are passively received from those that voluntarily choose to report any AEFI, and anyone can sign a report [16].

COVID-19 AEFI are quite common: most of them are mild and transient side effects, such as pain at the injection site, headache, fatigue, pyrexia, and chills, while severe reactions are incredibly rare [17]. The surveillance of COVID-19 AEFI is particularly challenging, since it involves a very large population in a short period of time [18].

According to the World Health Organization (WHO), there have been more than 430 million confirmed cases of COVID-19, and nearly 6 million deaths worldwide. As of 21 February 2022, a total of 10,407,359,583 vaccine doses have been administered [19].

The undeniable consistency of these numbers forces us to consider as accurately as possible the audiovestibular cost/benefit ratio associated with the largest vaccination campaign of all time.

The purpose of this study is to critically evaluate the consistency of the reports, analyze their scientific value, express plausible supporting theories, and possibly identify a shared pathway for the management of these patients.

## 2. Materials and Methods

A PubMed search was performed using the following keywords: “hearing loss”, “vertigo”, “dizziness”, “vestibular”, “otologic”, “deafness”, and “tinnitus” bound by the Boolean operator “AND” with the keyword “vaccine”.

The search period was restricted to articles produced from the date of marketing of the first COVID-19 vaccines until the date of submission (from early January 2021 to mid-February 2022).

The search produced a list of 112 articles. Articles that dealt with the topics in an anecdotal or marginal way were subsequently eliminated; duplicates were also eliminated, as well as reports without reliable instrumental evaluations. Consequently, the number of scientific articles considered for the drafting of this paper was reduced to 11.

The two main authors analyzed the articles (D.P. and F.M.G.), identifying the salient points and noting the data on a table. Any discrepancies were solved through discussion with a third author (P.V.), who subsequently produced the comparison and merger of the two works in the present final draft.

We considered as inclusion criteria for probable vaccine-related audiological adverse events every report of SSNHL, tinnitus, vertigo, and dizziness occurring within one month after vaccination.

SSNHL is defined as a sensorineural hearing loss of 30 dB or greater over at least three contiguous audiometric frequencies occurring within a 72-h period [20].

We considered reports of cases evaluated by a specialist or at least with a clear documentation of the hearing loss/vestibular deficit leading to a clear audio-vestibular diagnosis and subsequent therapy.

We established that two elements were essential to the diagnostic path: the brain imaging insights, visualized through computed tomography (CT) scan and magnetic resonance imaging (MRI), with MRI considered as the gold standard; and a negative reverse transcription polymerase chain reaction (RT-PCR) for SARS-CoV-2, via nasopharyngeal swab. Therefore, we critically evaluated the reports without this essential information.

Any reports leading to an alternative diagnosis for hearing loss (stroke, neurinoma, drug ototoxicity) were excluded.

Finally, we critically evaluated reports in subjects who previously experienced hearing loss, tinnitus, and vestibular disorders, but we did not exclude them from evaluation.

## 3. Results

The literature search for post-vaccine COVID-19 tinnitus as an isolated symptom was a complex task, due to the large number of articles where “tinnitus” was mentioned. Reports lacking diagnostic investigations and questionnaire-based papers were discarded. Only two studies passed the selection and were evaluated. Similarly, the search for the “vertigo” key also left few articles to evaluate. A larger sample has been linked to the sudden hearing loss variable; unfortunately, only few articles had data collected with objective scientific criteria.

The data regarding SSNHL and tinnitus as COVID-19 AEFI are collected in Table 1.

Wichova et al. [21] published an interesting data collection concerning the audiological manifestations following COVID-19 vaccination with the Moderna or Pfizer vaccine; through the identification of 30 patients, (mean age 60.9 ± 13.8 years old), it was found that the mean time of symptom onset was 10.18 ± 9 days post vaccination (range: 1 to 42 days). Overall, 25 patients (83.3%) complained of hearing loss, 15 (50%) of tinnitus, 8 (26.7%) of dizziness, 5 (16.7%) of vertigo, and 9 (30%) had other symptoms. These patients were treated with steroids (oral and intratympanic in some cases) and/or antivirals. All patient received a pure tone audiometry (PTA) and word recognition score (WRS) assessment; the authors reported that an MRI was requested for each patient, but there is a lack of MRI data, as well as post-treatment and follow-up evaluations.

Tsetsos et al. [22] in August 2021, reported a SSNHL, without tinnitus and vertigo, two days after the second dose of the AstraZeneca COVID-19 vaccine. The patient was a 61-year-old female with a history of hypertension, dyslipidemia, and thyroiditis, under pharmacological treatment long-term. The PTA showed profound pantonal right-SSNHL. She received steroid and acetylsalicylic acid treatment, leading to an almost full recovery within 15 days. MRI and MRA (magnetic resonance angiography) test were normal, and a RT-PCR test was not performed.

Jeong et al. [23] collected clinical data from three patients with SSNHL within two days after vaccination, of which two experienced severe hearing loss, one of whom recovered following therapy. The third patient had a partial recovery from his moderate hearing loss, but he underwent an audiological evaluation two weeks after the onset of the symptoms. Overall, two patients underwent MRI, which was normal, and RT-PCR testing was not performed.

The case of a healthy adult male with no previous ear problems who developed mild SSNHL two days after the first dose of the AstraZeneca vaccine was described by Pisani et al. [24] The patient underwent a complete evaluation, with blood chemistry tests, PTA, imaging tests, and RT-PCR testing. The treatment was started two weeks after the onset of SSNHL, leading to a partial recovery.

In total, five cases of SSNHL and one of vestibular neuritis following the AstraZeneca vaccine were described by Canales Medina et al. [25]. Among these, two cases did not meet the criteria for the diagnosis of SSNHL.

The sixth case was a vestibular neuritis with intense symptoms, promptly treated with a satisfactory recovery within seven days. One week later, the patient was treated for benign paroxysmal position vertigo (BPPV).

All patients described by these authors did not undergo RT-PCR or imaging tests.

A Sinovac AEFI was described by Zhao et al. [26] in January 2022. The authors collected two reports of severe SSNHL with tinnitus and dizziness, occurring four days after receiving the single dose of the vaccine. The patient underwent CT and MRI, which were normal. RT-PCR testing was not performed. They received a psychological evaluation, which excluded emotional instability. Unfortunately, their response to treatment was minimal.

Kahn et al. [27] presented the singular case of a 20-years-old male who developed multisystem inflammation and organ dysfunction beginning a few hours after the first dose of Pfizer COVID-19 vaccine. His clinical condition worsened in the next 48 h, with systemic inflammation, hematuria, progressive acute kidney failure (which required hemodialysis), and profound SSNHL. The patient had no history of hearing loss. During the diagnostic pathway, an acute multifocal ischemic stroke, pericardial and pleural effusion that required evacuation, and systemic capillary leak were discovered.

Expanded laboratory diagnostic evaluation was negative for autoimmune diseases but revealed a systemic dysregulated inflammatory process. Head and neck MRA did not suggest central nervous system (CNS) vasculitis as source of ischemic foci, while a non-contrast head MRI revealed multifocal punctate strokes in the brain, involving the left cerebellum. This distribution did not explain the SSNHL. Labyrinthitis and masses in the internal auditory canals were excluded by combining CT and MRI imaging.

After the failure of steroid therapy, the patient was candidate for cochlear implant. An RT-PCR test for SARS-CoV-2 was not performed.

Similar to the search concerning tinnitus, the variable “dizziness” generated a lot of candidates. However, most of them were lacking a clinical evaluation, or not related to the audiovestibular system. Data of tinnitus as COVID-19 AEFI are detailed in Table 2.

Tseng et al. [28] reported a case of new onset tinnitus, following the first dose of the AstraZeneca COVID-19 vaccine in a normal hearing subject who never previously experienced tinnitus. Blood testing excluded infections, and d-dimer and partial thromboplastin time were normal. The tinnitus handicap inventory (THI) quickly increased from 28 to 46, and the PTA was normal, while the short increment sensitivity test showed a high increment. The patient was subsequently treated with steroids, leading to a complete resolution of the symptomatology. The COVID-19 RT-PCR test revealed negative findings among the entire course of treatment, and MRI was not performed.

Parrino et al. [29] provided one of the first descriptive report, along with instrumental examinations, to document three cases of tinnitus following Pfizer vaccine injection. All patients received an audiological evaluation, PTA, and tinnitus test battery; however, only two patients underwent MRI. All patients denied a previous SARS-CoV-2 infection, and an RT-PCR test to check SARS-CoV-2 infection status was not performed. Patients’ history of previous pathologies related to the dysregulation of the immune system makes it possible to recognize a similar genesis for this new onset tinnitus. One of the patients (case 2) suffered from depression. In total, two patients had a positive response to steroid treatment, and one refused it (case 2), obtaining an improvement of the tinnitus regardless.

In December 2021, Jeong [30] described a case of vestibular neuritis (VN) in a 54-year-old male without previous audiological pathologies, which arose two days after inoculation with the COVID-19 Pfizer vaccine. Vestibular deficit was assessed with a video head impulse test (v-HIT), but the authors did not state whether imaging or RT-PCR testing was performed. The patient received a vestibular suppressant treatment and subsequent vestibular rehabilitation, gaining a complete clinical and instrumental recovery.

Recently Di Mauro et al. [31] evaluated 33 patients with “acute vertigo” after COVID-19 vaccination, including vertigo or dizziness appearing within 48 h from the inoculation. Among them, 16 reported objective vertigo, 14 subjective vertigo, and 3 dizziness. All patients underwent a meticulous and complete vestibular evaluation, which allowed the authors to exclude 7 patients, due to the absence of nystagmus, and 9 patients with BPPV; the remaining 17 cases were further referred to a neurologic assessment.

## 4. Discussion

The adverse effects of vaccines are mostly mild and self-limiting; pain at the injection site, redness or swelling at the injection site, fever, lymph node inflammation, headache, myalgias, joint pain, fatigue, and chills are the most common reported events. [17].

Most of the current vaccines against SARS-CoV-2 make use of the genetic code of the virus’ spike protein to stimulate a protective immune reaction. The adenovirus used in viral vector vaccines (Vaxzevria, Oxford-AstraZeneca, UK; Sputnik, Gamaleya Research Institute of Epidemiology and Microbiology, Russian Federation; Janssen, Johnson & Johnson, USA) incorporates the spike protein gene into its DNA, inducing S-protein formation, which leads to antibody production; this process confers protection against the virus. Conversely, mRNA vaccines (Comirnaty, Pfizer-BioNTech, USA-Germany; Spikevax, Moderna) allow the mRNA for spike protein to enter the host cells, stimulating an intense immune response. Other COVID-19 vaccines (BBIBP-CorV, Sinopharm, China; CoronaVac, Sinovac Biotech, China) use weakened or attenuated forms of the virus, which can still replicate, but not sufficiently to cause the disease itself in immunocompetent hosts [32].

The mRNA vaccine can elicit a self-adjuvating mechanism, causing the mRNA to act as both antigen and adjuvant at the same time. This could trigger an autoimmune reaction resulting in type I interferon production, which leads to a strong T- and B-cell response and an autoreactive lymphocytes activation [33].

A cross-reactivity, due to the phenomenon of molecular mimicry, between anti-spike SARS-CoV-2 antibodies and ear antigens is a possibility that could link COVID-19 vaccines to audiovestibular AEFI [34,35].

Audiovestibular COVID-19 AEFI are quite uncommon, but the literature shows growing evidence that SSNHL, tinnitus, and vertigo can be observed after immunization.

Despite this, only a few articles passed our inclusion criteria. This highlights poor attention to the timely collection of clinical data, together with objective instrumental evaluations.

Among the selected papers, seven were related to SSNHL following COVID-19 immunization, mainly with associated tinnitus [21,22,23,24,25,26,27].

Several reports of SSNHL after immunization with commonly used vaccines, such as rabies, hepatitis B, measles, and H1N1, are currently reported in the literature [36,37,38,39,40,41].

The estimated incidence of SSNHL is between 5 and 30 cases per 100,000 [42]. Interestingly, Wichova et al. [21] found an SSNHL incidence of 3.85% among patients evaluated during their clinical practice in 2021. These authors shared their retrospective data, affirming that SSNHL increased from 2019 to 2021, proposing the possibility of an association between COVID-19 or COVID-19 vaccination and SSNHL. However, the causality of this relationship remains unverified. The authors failed to specify the grade of SSNHL, as well as the response to treatment. Moreover, they found an interesting concordance between the onset of symptoms and the development of post-vaccine immunoglobulin G (IgG). The time frame may coincide with the onset of IgG production, which usually appears 10 to 14 days after priming [43].

This led the authors to hypothesize a possible autoimmune aetiology, according to the mechanism of cross-reaction. This hypothesis has been further corroborated by the collection of clinical data on patients with autoimmune diseases diagnosed before the vaccine which worsened following the vaccine. Moreover, the mean time of symptom onset did not change when comparing with a sub-group of patients with previous autoimmune inner ear disease (AIED) and Meniere’s disease (MD) diagnoses.

A limitation of this thesis is the absence of RT-PCR testing which could rule out COVID-19 infection in these patients. In fact, if not clearly excluded from a negative nasopharyngeal swab, there is a reasonable suspicion that symptoms are due to a new SARS-CoV-2 infection, which is known to be characterized by high neural tropism with potential damage to the inner ear, even in mild forms [44,45,46].

Similar to the viral effect on neural pathways of the olfactory sense [47], vertigo or hearing loss are known sequelae of viruses. Goddard et al., for example, found herpes simplex virus (HSV) DNA in the vestibular nerve fibres of patient suffering from VN [48].

The spectrum of neurologic syndromes caused by COVID-19 includes encephalitis, demyelination, and Guillain-Barré syndrome [49]. These pathologies could have a rationale even as AEFI, likely related to processes of vaccine vector particle dissemination in the tissues and through the blood circulation, as well as through the alteration of the blood–brain barrier in case of intense inflammatory reactions [50].

Tsetsos et al. hypothesized a thrombotic aetiology (or vasospasm) of the SSNHL case they reported, which was an adult female with multiple cardiovascular risk factors [22].

Dysregulation of the cochlear blood flow due to altered plasma viscosity, cellular and platelet aggregability, red blood cell deformability, and endothelial function have all been observed in patients affected by SSNHL [51].

Therefore, it is likely that these events will occur in the older population who get vaccinated, representing the result of a physio-pathological phenomenon that naturally takes place, rather than being direct consequence of vaccine administration [52].

Chen et al. postulated that the viral antigen–antibody complex triggers a hypersensitivity reaction, which can lead to a localised inflammation that damages the inner ear microvessels [53].

The SARS-CoV-2 spike protein is an effective activator of the complement alternative pathway, which may contribute to endothelial damage, and is an enhancer of platelet aggregation, leading to thrombus formation [54].

Sessa et al. analysed the reports of thromboembolic events following vaccination with the Pfizer-BioNTech or Moderna COVID-19 vaccines, compared to hormonal contraceptive use (known to be associated with an increased but accepted risk of thromboembolic events), and found a much higher safety profile in vaccines [55].

Another useful consideration is the good response to treatment; the responsibility of micro-thromboembolism due to COVID-19 vaccines would be less likely, since steroids do not have any anti-thromboembolism effects.

Among the possible causes favouring SSNHL, it is also necessary to mention those of a genetic nature, both in syndromic and non-syndromic forms [43,56]. They could be linked to increased susceptibility to ototoxic drugs known to be used during the pandemic [57,58].

The aetiopathogenesis of tinnitus is still debated. In our literature review, we noted that well-documented cases of tinnitus without hearing loss are rare. This seems to comply with a cortical reorganization due to sensorial deprivation as the most probable cause of tinnitus [59].

The cause of a possible increase in tinnitus during the pandemic may be increased anxiety and stress, since they share the same brain functional areas assigned to adaptive responses to sound stimuli [60]. Anzivino et al. postulated that an immunization anxiety-related reaction can be expected: anxiety has also been related to the severity and persistency of tinnitus, and it was found to be reasonable that the absence of environmental masking sound from everyday life should increase tinnitus perception [61].

Ueda et al. found a 45% reduction in the total number of vertigo-associated outpatient visits when comparing the period March–May 2020 with the same period of the 2019 [62].

Lovato et al. found a higher incidence of MD diagnosed for the first time, during the COVID-19 pandemic; moreover, known MD patients increased the number of vertigo attacks and reported higher dizziness handicap inventory (DHI) values, as compared to the previous year. These findings could be easily associated with the stress and higher anxiety present during the pandemic but could also relate to factors that could activate or boost the pathogenic mechanism of MD [63].

Vertigo and dizziness are two phenomena which are difficult to analyse in this review, since they are symptoms that are often reported without audiovestibular pathologies.

Few studies have been carried out with sufficient scientific accuracy, including Di Mauro et al., which had extreme difficulty in trying to homogenize a heterogeneous and small sample [31].

One important limitation we found in almost all screened papers was the lack of reporting of previous SARS-CoV-2 infection in the patients under study, or an incomplete pharmacological history. Moreover, only 2 studies out of 11 included RT-PCR testing to rule out possible SARS-CoV-2 infection.

These uncertainties that emerge from the literature could generate vaccine hesitancy and reluctance in the population. Surprisingly, in some cases, a certain hesitation or mistrust towards the vaccination campaign has been expressed even by health care workers, revealing an irrational fear, even in a professional category notoriously accustomed to experimental verification, towards the calculation of the cost/benefit, characterized by high resilience, especially during the pandemic [64,65].

Lastly, we suggest considering the emotional aspects of the pandemic and immunization campaign. As most of the reports in the major reporting systems are anecdotal, some of them are surely related to psychological and emotional aspects. The WHO defined the “anxiety-related adverse events following vaccination” as a “range of symptoms and signs that may arise around immunization that are related to anxiety and not to the vaccine product, a defect in the quality of the vaccine or an error of the immunization program” [66]. The Immunization Division of the Indian Ministry of Health and Family Welfare calculates that anxiety-related events account for up to 25% of total reports, including vasovagal-mediated reactions, hyperventilation mediated reactions, and stress-related psychiatric reactions or disorders [67].

This finding correlates with Zhang et al., who found that the most common AEFIs reported in a double-blind placebo-controlled clinical trial were pain, fever, and fatigue in the first 14 days after injection, both in the vaccination and placebo groups [68].

To date, none of the evaluated papers have unequivocally demonstrated a causal link between the COVID-19 vaccine and audiovestibular AEFI.

What the review of the literature makes apparent is the poor systemization of the data, which makes it difficult to aggregate cases, weakening the statistical power of the proposed theses.

All suspected COVID-19 AEFIs should be addressed to a specialist for rigorous audiovestibular assessment, and every report should be verified to collect as much standardized data as possible to increase our knowledge and increase the performance of systematic vaccine safety studies.

A proper identification, case study, and complete report is mandatory for enhancing the ongoing safety monitoring and supporting advances in mechanistic understanding.

Overall, the AEFIs are clearly outweighed by vaccine’s beneficial effects in decreasing hospitalization, mortality, and morbidity related to SARS-CoV-2 infection [69].

Our hope is that, through greater attention to the quality of data collection, we can reach a deeper knowledge of the audiovestibular AEFI. A big step forward would be to standardize the parameters to achieve greater statistical solidity.

## 5. Conclusions

Audiological and vestibular COVID-19 AEFIs are recognized events in scientific literature. This study underlines the possibility of acute inner ear damage after COVID-19 vaccination, which can be expressed as SSNHL, tinnitus, dizziness, vertigo, or a combination of them. The incidence appears rare, compared with the number of vaccines administrated.

There is a lack of systematization and standardization in the collection of clinical information, as well as in patient management. This makes it difficult to aggregate the cases and draw unambiguous conclusions.

To date, there is no evidence of an increased risk of vaccination-related audiovestibular events, nor is there evidence of a causal link.

## Figures and Tables

**Table 1:** SSNHL as COVID-19 adverse events following immunization (AEFI).

Authors	Age	Sex	Vaccine	Dose	Days to Onset of Symptoms	Hearing Loss Grade (PTA)	Response to Treatment	MRI	Tinnitus	Vertigo/Dizziness	RT-PCR
Wichova et al. [21]	74	F	Moderna	1	7	n/a	n/a	n/a	n/a	n/a	n/a
	73	M	Moderna	Both	3	n/a	n/a	n/a	n/a	n/a	n/a
	53	F	Pfizer	1	10	n/a	n/a	n/a	n/a	n/a	n/a
	51	M	Pfizer	1	14	n/a	n/a	n/a	n/a	n/a	n/a
	83	M	Moderna	2	10	n/a	n/a	n/a	n/a	n/a	n/a
	77	F	Moderna	2	30	n/a	n/a	n/a	n/a	n/a	n/a
	69	M	Pfizer	1	7	n/a	n/a	n/a	n/a	n/a	n/a
	67	F	Pfizer	Both	8	n/a	n/a	n/a	n/a	n/a	n/a
	60	M	Pfizer	2	10	n/a	n/a	n/a	n/a	n/a	n/a
	55	M	Pfizer	1	12	n/a	n/a	n/a	n/a	n/a	n/a
	54	F	Moderna	2	18	n/a	n/a	n/a	n/a	n/a	n/a
	49	M	Moderna	1	4	n/a	n/a	n/a	n/a	n/a	n/a
	43	M	Moderna	1	14	n/a	n/a	n/a	n/a	n/a	n/a
	86	M	Pfizer	2	42	n/a	n/a	n/a	n/a	n/a	n/a
	78	F	Pfizer	2	1	n/a	n/a	n/a	n/a	n/a	n/a
	76	M	Moderna	2	14	n/a	n/a	n/a	n/a	n/a	n/a
	67	M	Moderna	2	7	n/a	n/a	n/a	n/a	n/a	n/a
	66	F	Pfizer	2	7	n/a	n/a	n/a	n/a	n/a	n/a
	64	M	Moderna	2	7	n/a	n/a	n/a	n/a	n/a	n/a
	61	F	Pfizer	1	12	n/a	n/a	n/a	n/a	n/a	n/a
	59	M	Moderna	1	6	n/a	n/a	n/a	n/a	n/a	n/a
	58	F	Pfizer	1	10	n/a	n/a	n/a	n/a	n/a	n/a
	51	F	Moderna	Both	21	n/a	n/a	n/a	n/a	n/a	n/a
	48	M	Moderna	1	2	n/a	n/a	n/a	n/a	n/a	n/a
	39	M	Moderna	1	12	n/a	n/a	n/a	n/a	n/a	n/a
Tsetsos et al. [22]	61	F	AstraZeneca	2	2	severe	CR	normal	n/a	n/a	n/a
Jeong et al. [23]	64	F	AstraZeneca	1	1	severe	CR	normal	n/a	n/a	n/a
	42	M	Pfizer	1	1	mild	PR	n/a	n/a	n/a	n/a
	18	M	Pfizer	2	2	severe	worsened	normal	n/a	n/a	n/a
Pisani et al. [24]	57	M	AstraZeneca	1	2	mild	PR	normal	yes	no	negative
Medina et al. [25]	44	M	AstraZeneca	2	18	moderate *	CR	n/a	yes	n/a	n/a
	39	M	AstraZeneca	1	11	moderate	CR	n/a	yes	n/a	n/a
	43	M	AstraZeneca	2	14	moderate/severe	no	n/a	yes	n/a	n/a
	40	F	AstraZeneca	1	21	no	CR	n/a	no	yes	n/a
Zhao et al. [26]	30	M	Sinovac	1	4	severe	IR	normal	yes	dizziness	n/a
	64	F	Sinovac	1	4	severe	IR	normal	yes	dizziness	n/a
Kahn et al. [27]	20	M	Pfizer	1	2	profound	no	focal	yes	n/a	n/a

Data from Di Mauro et al. are not represented here, since most clinical data are aggregated. CR (complete recovery), PR (partial recovery), IR (insufficient recovery). * (bilateral hearing loss), focal (MRI showed focal lesions)

**Table 2:** Tinnitus as COVID-19 adverse events following immunization (AEFI).

Authors	Age	Sex	Vaccine	Dose	Onset: Days (h)	Hearing Loss Grade (PTA)	THI before Treatment	Response to Treatment	THI after Treatment	RT-PCR	MRI
Ping-Tao Tseng et al. [28]	37	M	AstraZeneca	1	0 (5 h)	no	28–46	yes	0	neg	n/a
Parrino et al. [29]	37	F	Pfizer	1	0 (7 h)	no	90	no	78	n/a	normal
	63	M	Pfizer	1	0 (20 h)	Mild (before vaccine)	76	refused	36	n/a	n/a
	30	M	Pfizer	2	7	no	78	yes	6	n/a	normal

## Data Availability

Not applicable.
